# Microplastics and Nanoplastics: The Next Frontier of Cardiovascular Risk?

**DOI:** 10.1016/j.jacadv.2024.101510

**Published:** 2024-12-20

**Authors:** Robert O’ Dowling

**Affiliations:** Tallaght University Hospital, Dublin, Ireland

**Keywords:** cardiovascular disease, emerging cardiovascular risk factors, microplastics, nanoplastics, MNPs

Cardiovascular disease is a group of diseases affecting the heart and blood vessels, it accounts for 30% of all deaths worldwide.^1^ The concept of risk factors for cardiovascular disease was initially proposed in 1951 following the findings of the Framingham study. Dawber et al published their seminal paper “Epidemiological approaches to Heart Disease: The Framingham Study” in the *American Journal of Public Health and the Nation's Health*.^2^ We have both the authors of this research as well as the people of Framingham, a town 20 miles west of Boston, to thank for what ultimately led to a 50% reduction in mortality due to cardiovascular disease in the United States. The results from Dawber et al’s study lead to a significant shift in public health policy as well as transforming the conventional approach to the management of cardiovascular disease, which prior to the Framingham study was the cause for half of the deaths in America.^3^

Dawber et al were the first to identify the role hypertension, smoking as well as obesity play in the progression of ischemic heart disease.^2^ The prevention of cardiovascular disease has relied on these findings ever since. As society evolves in both an industrial and technological sense, so too has our exposure to different plastics and microplastics which, in previous animal studies, have been proven to have a toxic effect on the heart.^4^ The global production of plastics continues to rise dramatically. In 1950, the world produced just 2 million tonnes of plastic; it now produces 450 million tonnes.^5^ Given the vastly increased production and potential for cardiotoxicity, is it time to consider microplastics and nanoplastics (MNPs) a novel risk factor for cardiovascular disease?

Microplastics are plastic fragments with a particle size of <5 mm, while nanoplastics are typically considered to have a particle size of <1 μm.^6^ Primary MNPs are particles that are intentionally manufactured with micron or nanoscale dimensions while secondary MNPs are derived from the degradation by-products of large plastic debris. The use of plastics in society is ubiquitous and is notably present in medical, cosmetic, and food products.^4^ There are a number of means by which MNPs enter the human body including oral exposure, inhalational exposure as well as dermal contact.^4^ It seems that MNPs have a predilection for highly vascular structures and have been identified in the heart, lungs, and placenta of humans.^6^

Previous animal studies have raised concern over the toxic effect of MNPs on the cardiovascular system.^4^ There has been a range of proposed mechanisms of toxicity including oxidative stress, inflammation, apoptosis, and pyroptosis. Through these proposed mechanisms, MNPs have been associated with insults to the vasculature in the form of vascular endothelial damage as well as dysregulation of blood coagulation, thrombosis and hemolysis. The proposed cardiotoxic effects of MNPs have previously been associated with abnormal heart rates, impaired cardiac function, pericardial edema and myocardial fibrosis.

In terms of the cardiotoxic effects of MNPs, it is worth noting that the research to date, while seemingly displaying a causal relationship between MNPs and dysregulated cardiac function has at times proven contradictory. An example of this being the effects MNPs have on heart rate.^4^ Exposure to MNPs was noted to cause a dose-dependent reduction in the heart rate of zebrafish while also causing an increased heart rate in goldfish larvae. One potential explanation of this being the size and type of the MNP tested as well as the exposure dose and duration used in different studies.

A recent study by Marfella et al^6^ in the *New England Journal of Medicine* is the first observational study that has reported an association between MNPs and adverse cardiovascular events. Marfella et al^6^ tested the excised atheroma from patients undergoing carotid endarterectomy for the presence of MNPs by means of pyrolysis chromatography mass spectrometry. They subsequently reported that patients with MNPs present in the atheroma sampled from their carotid artery had a statistically significant higher incidence of a composite of nonfatal heart attack, nonfatal stroke, or death during follow up. Marfella et al^6^ have also identified that higher concentrations of typical markers of inflammation that promote the progression of atherosclerosis were noted in the cohort of patients that had MNPs detected in their carotid atheroma.

Given the in vivo and in vitro evidence to suggest that MNPs produce cardiotoxic effects, as well as the recent study by Marfella et al,^6^ identifying an association between the presence of MNPs in atheroma and subsequent cardiovascular events, are we now forced to consider MNPs as a novel risk factor for cardiovascular disease?

The Marfella et al^6^ paper has undoubtedly ignited an interest in this topic for clinicians, however, it is worth noting that while this study does report an association between MNPs and cardiovascular risk, it is not possible to extrapolate a causal relationship between the presence of MNPs and subsequent cardiovascular events. The authors were commendably transparent in the reporting of their study design and results. This is the first study of its kind to report an association between MNPs and cardiovascular events; however, there are some notable caveats in relation to the study design and subsequent outcomes.

Firstly, in the Marfella et al^6^ study, they reported the incidence of the composite endpoint (nonfatal stroke, nonfatal myocardial infarction, and death) in the group of patients that had MNPs noted in their atheroma (n = 150) versus those that did not (n = 107). The event rate was 30 of 150 in the first group of patients (those with MNPs noted in their excised atheroma) and 8 of 107 in the second group. In the supplementary appendix, the authors outline the breakdown of nonfatal stroke, nonfatal myocardial infarction, and death within the composite endpoints for each group. Of the 30 patients in the group with MNPs noted in their excised atheroma who reached the primary endpoint, 14 were due to stroke (47%). 5 of 8 (62.5%) of patients in the group without evidence of MNPs reached the primary endpoint of nonfatal stroke. In a cohort of patients that are undergoing endarterectomy, whereby the previous literature has reported bilateral carotid artery stenosis being present in 68% of cases,^7^ is it possible to generalize that the participant’s subsequent cardiovascular risk (when stroke is the predominant outcome event) is attributable to the presence or absence of MNPs rather than their predisposed risk of stroke due to established carotid artery stenosis?

Secondly, it must also be noted that follow-up was defined as per standard postprocedure clinic reviews. The trial did not mandate specific assessment visits for the purposes of the study. The follow-up data were therefore derived from electronic patient records. 47 of 304 (15%) of those enrolled had incomplete data or were lost to follow-up. Of the 21 patients who were lost to follow-up by the clinical team, it would be of particular interest to know which group these patients belonged to as well as the reason for their loss to follow up. 15% of patients excluded from the trial is notably lower than the projected 20% loss to follow-up incorporated into the trial sample size calculation. However, it would seem suspicious that such a large proportion of patients would not attend follow-up after such significant surgery, raising the question over whether this figure is confounded by patient death, which would in turn influence the primary endpoint.

Thirdly, the Marfella et al^6^ study did not test for the presence of MNPs in the serum samples of patients included in the trial. This would be a particularly interesting addition as it would allow us identify whether the group of patients without MNPs detected in their atheroma did in fact have exposure to MNPs by means of their bloodstream. This would not only help answer the question as to whether environmental exposure was the difference between the 2 groups but it would also prompt consideration as to what physical characteristics of the carotid atheroma make it more susceptible to the accumulation of MNPs.

Finally, the risk of contamination of the excised specimens is not negligible in this instance as the surgical environment as well as surgical equipment used contain many MNPs and this may have posed a risk of contamination.^8^

Our understanding of the cardiotoxic effects of MNPs has predominantly been derived from animal studies. From a clinical perspective, investigation of the association between MNPs and cardiovascular outcomes has been spearheaded by Marfella et al. While there are many unanswered questions at present, “Pandora’s box” has undoubtedly been opened.

With this in mind, maybe it is time to once again return to the foundations of where cardiac risk was first proposed. Similar to how the authors of the Framingham study set out 80 years ago, is there now a place for a prospective, observational study enrolling healthy volunteers, testing their levels of MNPs in serum, and observing for future cardiovascular events? Only time will tell.

## Funding support and author disclosures

The author has reported that they have no relationships relevant to the contents of this paper to disclose.
